# Oncomirs Expression Profiling in Uterine Leiomyosarcoma Cells

**DOI:** 10.3390/ijms19010052

**Published:** 2017-12-25

**Authors:** Bruna Cristine de Almeida, Natalia Garcia, Giovana Maffazioli, Laura Gonzalez dos Anjos, Edmund Chada Baracat, Katia Candido Carvalho

**Affiliations:** Laboratório de Ginecologia Estrutural e Molecular (LIM 58), Disciplina de Ginecologia, Departamento de Obstetricia e Ginecologia, Hospital das Clinicas da Faculdade de Medicina da Universidade de Sao Paulo, HCFMUSP, SP, BR Av. Dr Arnaldo 455, sala 4121, 05403-010 Cerqueira Cesar, São Paulo, Brazil; bruc_10@hotmail.com (B.C.d.A.); natalia.garciaft@gmail.com (N.G.); giovanamaffazioli@hotmail.com (G.M.); lauragonzalezanjos@gmail.com (L.G.d.A.); edmund.baracat@hc.fm.usp.br (E.C.B.)

**Keywords:** uterine leiomyosarcoma, uterine leiomyoma, miRNA, oncomirs, cell culture

## Abstract

MicroRNAs (miRNAs) are small non-coding RNAs that act as regulators of gene expression at the post-transcriptional level. They play a key role in several biological processes. Their abnormal expression may lead to malignant cell transformation. This study aimed to evaluate the expression profile of 84 miRNAs involved in tumorigenesis in immortalized cells of myometrium (MM), uterine leiomyoma (ULM), and uterine leiomyosarcoma (ULMS). Specific cell lines were cultured and qRT-PCR was performed. Thirteen miRNAs presented different expression profiles in ULM and the same thirteen in ULMS compared to MM. Eight miRNAs were overexpressed, and five were underexpressed in ULM. In ULMS cells, five miRNAs exhibited an overexpression and eight were down-regulated. Six miRNAs (miR-1-3p, miR-130b-3p, miR-140-5p, miR-202-3p, miR-205-5p, and miR-7-5p) presented a similar expression pattern in cell lines compared to patient samples. Of these, only three miRNAs showed significant expression in ULM (miR-1-3p, miR-140-5p, and miR-7-5p) and ULMS (miR-1-3p, miR-202-3p, and miR-7-5p). Our preliminary approach identified 24 oncomirs with an altered expression profile in ULM and ULMS cells. We identified four differentially expressed miRNAs with the same profile when compared with patients’ samples, which strongly interacted with relevant genes, including apoptosis regulator (BCL2), epidermal growth factor receptor (EGFR), vascular endothelial growth factor A (VEGFA), insulin like growth factor 1 receptor (IGF1R),serine/threonine kinase (RAF1), receptor tyrosine kinase (MET), and bHLH transcription factor (MYCN). This led to alterations in their mRNA-target.

## 1. Introduction

MicroRNAs (miRNAs) are endogenous 17–27 nucleotide-long non-coding RNAs acting as regulators of gene expression at the posttranscriptional level by inhibiting protein synthesis. They play a key role in several biological processes [1], and, when abnormally expressed, may lead to cellular transformation and tumorigenesis. Approximately 50% of miRNAs are located in chromosomal regions associated with cancer [2]. They are considered to be oncomirs, which regulate oncogenes or tumor suppressor genes and exert functional effects on tumor cell signaling pathways [2,3,4]. Specific expression patterns were seen in different tissues, which suggests that they have potential utility as clinical biomarkers [2].

Uterine leiomyosarcoma (ULMS) is a smooth muscle [5] rare malignant tumor, representing about 1–2% of all uterine neoplasias [6,7]. It is associated with a worse prognosis even when diagnosed early. It has a recurrence rate of about 53–71% [8]. Main molecular features of this tumor includes genetic instability, loss of heterozygosity, and alterations in gene expression [9,10,11]. In addition, some miRNAs were previously identified as having altered expression in uterine leiomyoma (ULM) and in ULMS [12]. There is a hypothesis that ULM development may result in ULMS. Although the molecular mechanisms involved in this process are still unknown [6,11,13], differences in miRNA expression may play a role in this malignant transformation. Attempting to find diagnostic and prognostic biological biomarkers for this tumor, we performed an epigenetic study. The present study aims to evaluate the expression profile of 84 oncomir sequences in immortalized cells of myometrium (MM), ULM, and ULMS.

## 2. Results

Formalin-fixed paraffin-embedded (FFPE) tissue samples comprised 13 patients in the MM group (control group) with ages ranging from 39–55 years old (mean age: 46.46 ± 5.125 years old; data represents the mean ± SD) and race being 54% white and 46% non-white. The ULM group was composed for 10 white women with ages ranging from 31–51 years old (mean age: 42.9 ± 6.887 years old). The ULMS group had 16 patients with ages ranging from 27–84 years old (mean age: 54 ± 16.99 years old). In this group, 50% of patients were white and 12.5% were non-white, while 37.5% of women did not declare their ethnicity. Groups did not differ from the age mean (*p* = 0.1377). However, the difference in ethnicity was statistically significant between groups (*p* = 0.0351). Other clinical information from ULMS patient are described in Table 1.

**Figure 1:**
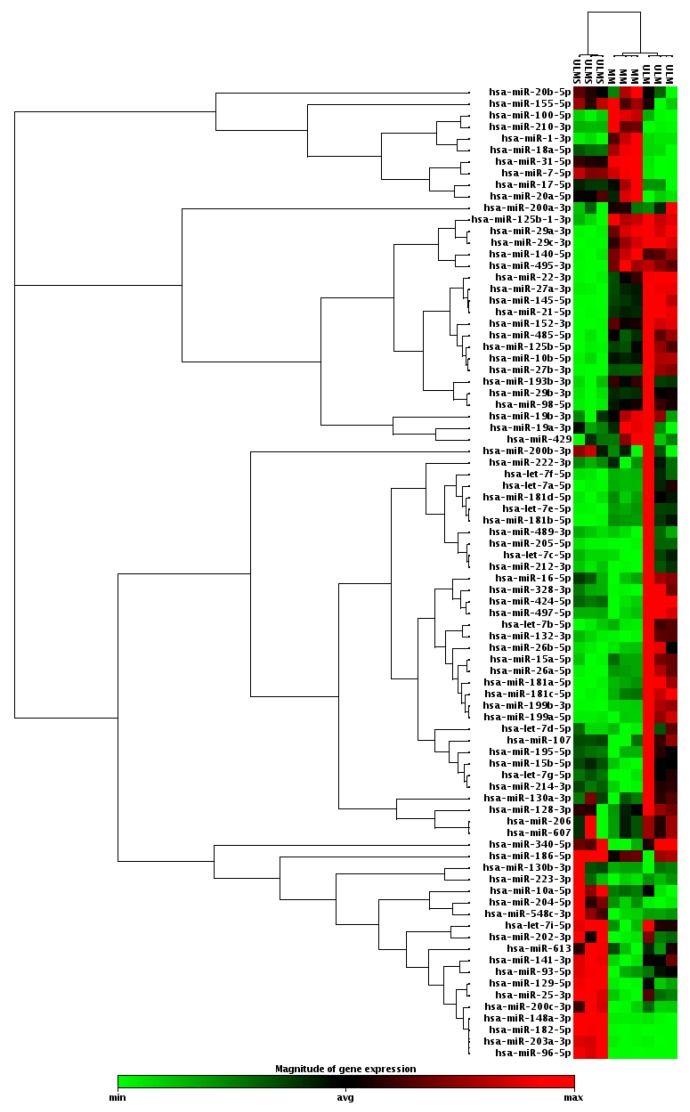
Clustergram analysis with profile expression of 84 oncomirs in uterine leiomyoma (ULM) and uterine leiomyosarcoma (ULMS) cells compared with myometrium (MM) cells (fold change (FC) expression cut-off values of +2 and −2).

In order to classify cell types according to their miRNA profiles, hierarchical clustering analysis was performed. The miRNA profile was able to cluster ULM, ULMS, and MM cells. Data analysis showed 37 miRNAs with significantly different expressions in ULM and 38 in ULMS when comparing to MM (Figure 1).

**Figure 2:**
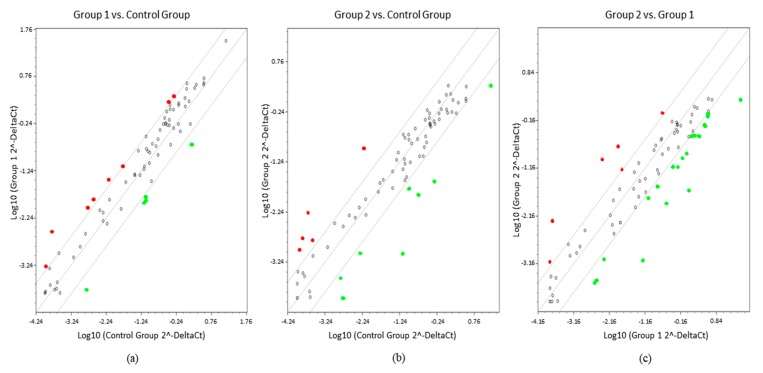
Scatter plots showing comparisons between groups. (**a**) ULM (group 1) compared with MM (control group); (**b**) ULMS (group 2) compared with control group; (**c**) Group 1 compared with group 2 (FC expression cut-off values of +4 and −4). In red, overexpressed miRNAs and, in green, underexpressed miRNAs.

In order to provide a more detailed investigation, the threshold line was increased from X to Y of −4 and +4. Elevation of the cut-off value distinguished 13 miRNAs with significant different expression in ULM compared to MM. Of these, eight were overexpressed (miR-132-3p, miR-199b-3p, miR-199a-5p, miR-202-3p, miR-205-5p, miR-212-3p, miR-328-3p, and miR-497-5p) and five were underexpressed (miR-1-3p, miR-130b-3p, miR-210-3p, miR-31-5p, and miR-7-5p). Thirteen altered miRNAs were found to be differentially expressed in ULMS compared to MM, where five were overexpressed (miR-129-5p, miR-141-3p, miR-148a-3p, miR-202-3p, and miR-203a-3p) and eight were underexpressed (miR-1-3p, miR-125b-1-3p, miR-140-5p, miR-152-3p, miR-21-5p, miR-27b-3p, miR-485-5p, and miR-495-3p). Comparative analysis between ULM and ULMS displayed six up-regulated miRNAs (miR-130b-3p, miR-148-3p, miR-203a-3p, miR-204-5p, miR-31-5p, and miR-7-5p) and 20 down-regulated (miRNAs let-7a-5p, let-7b-5p, let-7e-5p, miR-10b-5p, miR-125b-1-3p, miR-140-5p, miR-145-5p, miR-152-3p, miR-181a-5p, miR-181b-5p, miR-181c-5p, miR-199b-3p, miR-199a-5p, miR-205-5p, miR-21-5p, miR-22-3p, miR-27a-3p, miR-27b-3p, miR-29a-3p, miR-485-5p, and miR-495-3p) in ULMS cells (Figure 2). 

**Figure 3:**
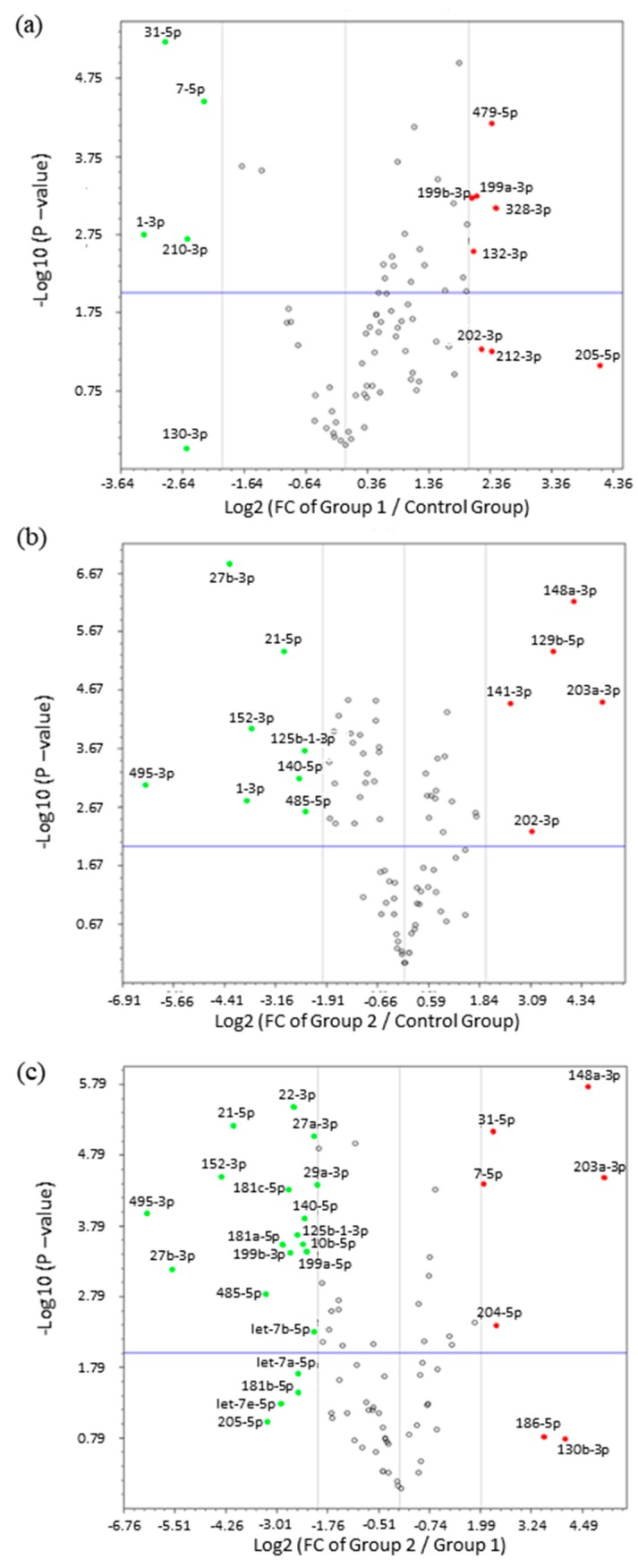
Volcano plots with differentiated expression of miRNAs. (**a**) miRNAs expression in ULM (group 1) compared with MM (control group); (**b**) miRNA expression in ULMS (group 2) compared with MM; (**c**) miRNAs expression in ULMS compared with ULM. FC expression cut-off values of +4 and −4. In green, down-regulated miRNAs and, in red, up-regulated miRNAs.

**Figure 4:**
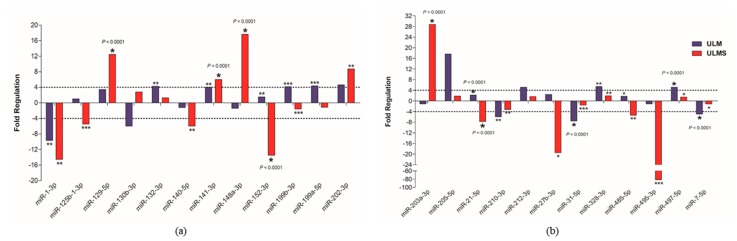
Analysis of 24 differentially expressed miRNAs. (**a**,**b**) Expression levels of cell lines and MM tissue, with the MM cell used as reference (cut-off +4 and −4). Student’s *t*-test; * *p* < 0.05, ** *p* < 0.005, and *** *p* < 0.0005 (the error bars were not provided by software).

Subsequent analysis of fold change by volcano plot identified 24 miRNAs that were highly deregulated in ULM and ULMS. The expression pattern of ULM evidenced that the miRNAs miR-31-5p and miR-7-5p were significantly underexpressed (*p* < 0.0001) while the miR-497-5p was overexpressed (*p* < 0.0001). In ULMS, 3 miRNAs—miR-21-5p, miR-27b-3p, and miR-152-3p—had a highly significant underexpression (*p* < 0.0001), and 4 miRNAs—miR-129-5p, miR-141-3p, miR-148a-3p, and miR-203a-3p—presented a significant overexpression (*p* < 0.0001) (as seen in Figure 3 and Figure 4). Differences in miRNA expression between ULM and ULMS were also assessed. Referring to the ULM cell line, 6 miRNAs were up-regulated and 20 were down-regulated in ULMS (as seen in Figure 3c).

**Figure 5:**
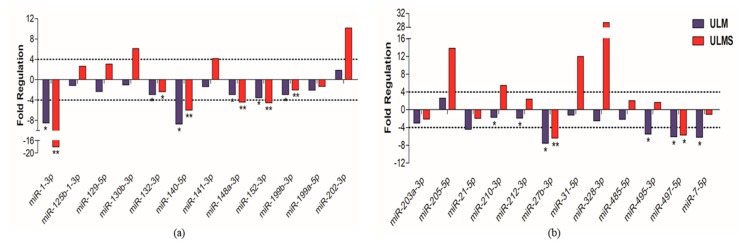
Analysis of 24 differentially expressed miRNAs in human samples. (**a**,**b**) Twelve miRNAs with significant expression in patients’ samples, having as reference MM tissue (cut-off +4 and −4). Student’s *t*-test; * *p* < 0.05, ** *p* < 0.005, and *** *p* < 0.0005 (the error bars were not provided by software).

**Figure 6:**
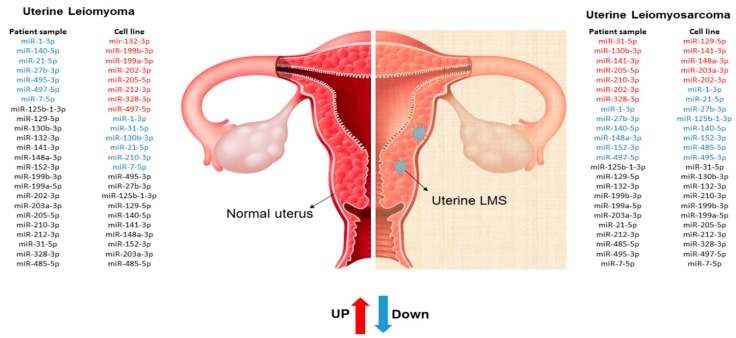
Schematic illustration of miRNAs expression profile in cell and patient samples.

Since cell line cultures may have some particularities that may not correspond to the same mechanisms found “in vivo”, the miRNA expression profile found in our cells culture was compared with human samples of MM, ULM, and ULMS. Assessment of miRNA expression in the cell culture and the patient samples showed that miR-1-3p, miR-130b-3p, miR-140-5p, miR-202-3p, miR-205-5p, and miR-7-5p had a similar pattern in the cell culture and human tissue. However, only the miR-1-3p was underexpressed in ULM (*p* = 0.0017) and ULMS (*p* = 0.0016) cells. The miR-7-5p showed a relevant down-regulation in ULM (*p* < 0.0001) while the miR-140-5p was significantly underexpressed in ULMS (*p* = 0.0006). In addition, the miR-202-3p was significantly overexpressed in ULMS cells (*p* = 0.0055) (as seen in Figure 4 and Figure 5). The miR-130b-3p, miR-205-5p, and miR-212-3p were not significant. Figure 6 is showing the miRNAs expression in short.

**Figure 7:**
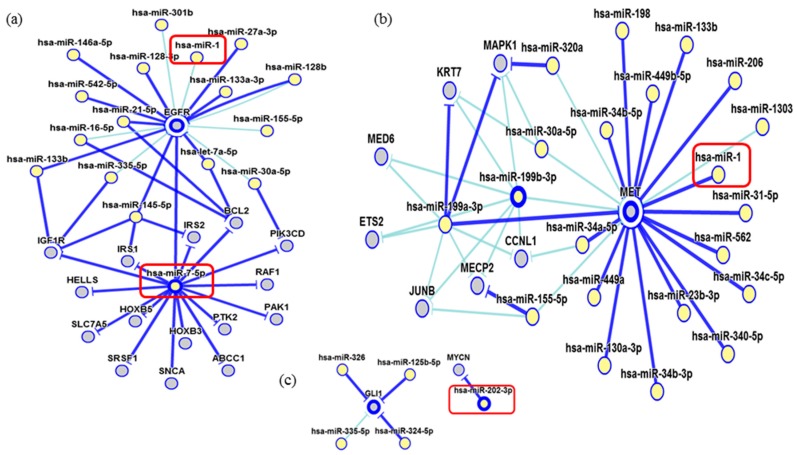
Interaction network of miR-7-5p and miR-1. (**a**) The miR-7-5p strongly modulates the EGFR, IGF1R, RAF1, and BCL2 genes while the miR-1 showed a weak interaction; (**b**) The miR-1 has a strong interaction with MET; (**c**) miR-202 showing a strong interaction with MYCN. In dark blue: strong evidence (reporter assay, Western blot, qRT-PCR, or qPCR) and, light blue, other evidence.

**Figure 8:**
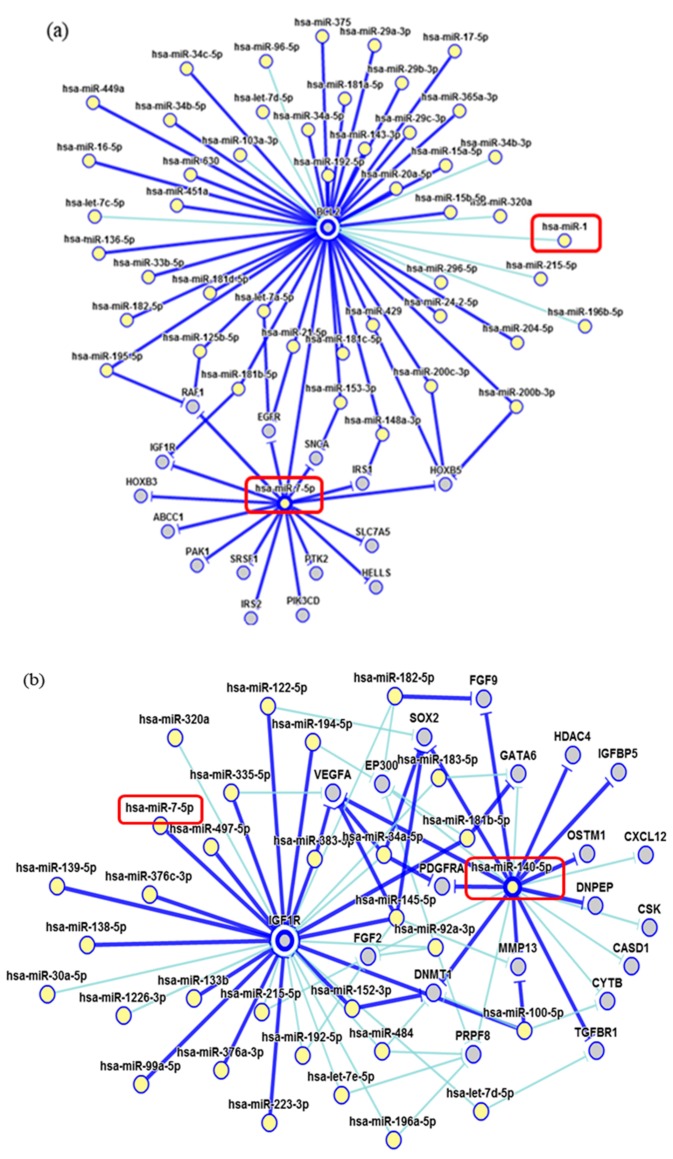
Interaction network of miRNAs. (**a**) miR-1 showed a weak modulation of BCL2; (**b**) miR-140-5p strongly interact with VEGFA. Dark blue shows strong evidence (reporter assay, Western blot, qRT-PCR or qPCR), and light blue shows other evidence.

**Figure 9:**
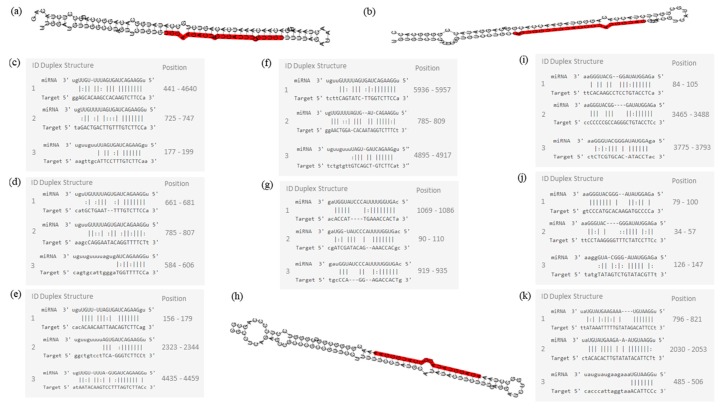
Molecular structures of miR-7-5p, miR-140-5p, and 202-3p. (**a**) Pre-miRNA hairpin of miR-7-5p, second structure of pre-miRNA, and, in red, mature sequence: 24| UGGAAGACUAGUGAUUUUGUUGU |46; (**b**) Pre-miRNA hairpin of miR-140-5p, 2nd structure of pre-miRNA, and, in red, mature sequence: 23| CAGUGGUUUUACCCUAUGGUAG |44; (**c**) miR-7-5p sequence of target interactions—EGFR; (**d**) miR-7-5p sequence of target interactions—RAF1; (**e**) miR-7-5p sequence of target interactions—BCL2 and position in the gene sequence; (**f**) miR-7-5p sequence of target interactions—IGF1R; (**g**) miR-140-5p sequence of target interactions—VEGFA; (**h**) Pre-miRNA hairpin of miR-202-3p, 2nd structure of pre-miRNA, and, in red, mature sequence: 64| AGAGGUAUAGGGCAUGGGAA |83; (**i**) miR-202-3p sequence of target interactions—IGF1R; (**j**) miR-202-3p sequence of target interactions–GLI1; (**k**) miR-1-3p sequence of target interactions—MET. Mature sequence of miR-1-3p: 53| UGGAAUGUAAAGAAGUAUGUAU |74. Pre-miRNA hairpin structure of miR-1-3p is unknown.

Network of genetic interactions of miR-7-5p, miR-1-3p, miR-140-5p, and miR-202-3p were described in Figure 7 and Figure 8. miRNAs molecular structures and miRNA-target interactions regions in Figure 9.

## 3. Discussion

This preliminary study found specific miRNAs signature patterns in uterine tumor cell lines. Out of the 84 human miRNAs on the array, we selected 24 relevant oncomirs. Expression values of 13 oncomirs were detected above the threshold levels in the myometrium and in both ULM and ULMS cells. In the same cell line used in our study, Kowalewska et al. [3] evaluated the fold changes in the miRNA expression in the uterine sarcoma (MES-SA) and uterine leimyosarcomas (SKUT-1) cell lines and compared them to healthy uterine tissue samples. They did not find significant differences between cells lines and human tissue. In this same study, the authors analyzed endometrial sarcomas, leiomyosarcomas, and mixed epithelial-mesenchymal tumors and compared them to control uterine tissue. Unlike our study, no significant changes in miRNA expression levels were found between leiomyosarcoma and control. However, Chuang et al. [10] found decreased expression of miR-200c in leiomyosarcoma cell lines (SK-LMS-1) compared to isolated ULM human cells.

We observed a greater number of up-regulated miRNAs in the ULM cell, while low expression was seen in almost all ULM patient samples. These differences in the profile may be explained by the high sarcoma heterogeneity [11,14]. Unfortunately, the limited number of samples in the present study did not allow correlation of the miRNA profile with different disease stages and mortality. Of note, tumors also alter cell type composition. Due to this, it is best to separate these effects in the profiling of heterogeneous tumor samples by characterizing tumor cell lines [4].

Interestingly, studies pointed out that miR-202 are down-regulated in many kinds of cancers, such as pancreatic cancer, colorectal, osteosarcoma, cervical, and endometrial adenocarcinoma [15,16,17]. Notwithstanding, our data presented a significant overexpression of miR-202-3p in ULMS, but not in ULM cells, suggesting a potential oncogenic result in the ULMS cells. This miRNA is a strong negative regulator of proto-oncogene MYCN expression, but its expression has not been reported in ULMS yet.

Although not established, some evidence suggests that the miR-202-3p may interact with the GLI1 gene and may be correlated with uterine smooth muscle tumors. In an immunohistochemical analysis, Garcia et al. [13] observed a gradual increase of GLI1 expression in ULM and ULMS cells when compared to normal MM. However, it is necessary to evaluate the GLI1 expression in cell lines to elucidate the direct and/or indirect effects of miR-202 overexpression while taking into account that immortalized cell line expression may present some differences in relation to FFPE tissues. Thus, detailed studies are necessary to understand this differential expression compared with other tumor types.

In the current study, two miRNAs presented a significant underexpression in ULMS cells and one ULM cell line (miR-1-3p, miR-140-5p, and miR-7-5p, respectively). This expression pattern was the same one found in human samples. The miR-1-3p seems to modulate critical genes as Bcl-2, which regulates apoptosis [18]. Estrogen receptor 1 (ESR1), which mediates ULM development and appears to be expressed in ULMS [19] and c-MET cells, acts in tumor dissemination by activating mitogenic signaling pathways [20]. Oncomir miR-140-5p strongly modulates the transcription of the growth factor VEGFA, which plays a role during ULM cell growth and may have higher levels in ULM tissue [21]. Another important gene directly regulated by miR-140-5p is the IGF1R, which has been detected in the uterus. Its levels of expression seem to be significantly higher in leiomyoma than in the myometrium cell. This gene acts as a survival factor that inhibits apoptosis and may increase its tumorigenic potential, which protects them from programmed cell death when over-expressed [22]. Moreover, miR-7-5p had a significant underexpression in our results in ULM cells. This miRNA regulates the EGF-R protein, which has been described as immunolocalized of its proteins in the cytoplasm of smooth-muscle cells in the ULM and matched MM [23] and Raf-1 cell lines, which play an important role in cellular antiapoptosis [24].

Tumor suppressor or oncogenic miRNAs are being considered targets for new therapeutic strategies. These new therapies are seeking to recover tumor suppressor proteins and inhibit oncogenic ones by restoring the miRNAs that are underexpressed and silencing the miRNAs that are up-regulated [25]. miRNA abnormality is well-tolerated in normal tissues, but it can profoundly influence the behavior of cells and tissues. Thus, miRNA inhibition or delivery may provide a highly potent means to modulate a disease process while avoiding unwanted toxic effects in normal tissues [26]. The expression profile of miRNAs may be relevant as a diagnostic biomarker for differentiating ULM cells from ULMS cells. These tumors have similar radiographic appearances, and ULMS cells are not diagnosed until the time of surgery. Due to the risk of accidental dissemination of the ULMS tissue during removal of a presumed benign uterine fibroid, the identification of a biomarker would facilitate referral to a proper initial surgical procedure [27].

In summary, our preliminary approach identified 24 oncomirs with a deregulated expression profile in ULM and ULMS. Of these, four oncomirs with the same profile both in cell lines and human samples might lead to alterations in their mRNA-target. Our results provide evidence that the simultaneous evaluation of miRNA expression in ULM and ULMS cells can add new information to the treatment strategy in uterine tumor development and treatment.

## 4. Materials and Methods

### 4.1. Cell Culture

ULM (THESCs CRL-4003), ULMS (SK-UT-1 HTB-114), and MM (PCS-460-011) cell lines were purchased from the American Type Tissue Collection (ATCC, Manassas, VA, USA). Cells were seeded in 75 cm^2^ bottles in DMEM/F-12-D2906-10L (Sigma-Aldrich, Darmstadt, Germany), Basal Medium Eagle-B9638-10X (Sigma-Aldrich, Darmstadt, Germany) and DMEM/F-12-D8900-10L (Sigma-Aldrich, Darmstadt, Germany) medium, respectively, and supplemented with 10% of fetal bovine serum (FBS) (Gibco, Carlsbad, CA, USA) and 100 U/mL penicillin, 100 µg/mL streptomycin, and gentamycin in 37 °C incubators with 5% CO_2_. 5 × 10^6^ cells were collected, washed in PBS, and stored at −20 °C.

DNA was extracted with DNeasy^®^ Blood and Tissue-kit (Qiagen, Hilden, Germany) and sequenced with GenePrint^®^ 10 System-kit (Promega, Madison, WI, USA) for authentication. All loci were confirmed in the ATCC database.

### 4.2. Human Samples

All subjects gave their informed consent for inclusion before they participated in the study. The study was conducted in accordance with the Declaration of Helsinki and the protocol was approved by the Research Ethics Committee of the Faculdade de Medicina da Universidade de Sao Paulo—FMUSP (number 1.517.306, 27 April 2016). In order to compare the miRNA expression profile of cell culture lines with human samples, total RNA was extracted from 39 formalin-fixed paraffin-embedded (FFPE) human samples stored at the Molecular and Structural Gynecology Laboratory of University of Sao Paulo Medical School (FMUSP).

### 4.3. MicroRNA and RNA Isolation

microRNAs were extracted from all cell lines using the mirVana™ miRNA Isolation-kit (Ambion, Foster City, CA, USA). The FFPE patients’ samples were extracted with ReliaPrep™ FFPE Total RNA Miniprep System (Promega, Madison, WI, USA). All samples were quantified by the spectrophotometer NanoDrop 2000 (Thermo Scientific™, Fremont, CA, USA) and reverse transcription was performed using the miScript II RT-kit (Qiagen, Hilden, Germany) according to manufacturer’s instructions.

### 4.4. microRNA qRT-PCR

Quantitative Real-Time PCR (qRT-PCR) was carried out using the miScript SYBR^®^ Green PCR-kit (Qiagen, Hilden, Germany) in triplicate with MIHS-109ZA-Qiagen 96 wells plate (Qiagen, Hilden, Germany). This kit contains 84 oncomir assays. Reactions were incubated for 15 min at 95 °C followed by 40 cycles for 15 s at 94 °C, 30 s at 55 °C, and 30 s at 70 °C. The level of miRNA expression was determined using SNORD61 and SNORD68 for normalization based on the geNormTM algorithm analysis software version 3.0 (Biogazelle, Zwijnaarde, Belgium) [28]. All data of relative expression were analyzed with the comparative cycle threshold method by ΔΔ*C*_t_ [29]. In silico analysis was performed to identify the genetic interaction network strongly related to tumorigenesis [30].

### 4.5. Statistical Analysis

All statistical analyses were performed using GraphPad Prism 5.0 (GraphPad Software, San Diego, CA, USA). Continuous data were analyzed for normality. Students’ *t*-test or ANOVA were used for between-group comparisons for parametric variables and Mann-Whitney U or Kruskal-Wallis for non-parametric variables. Pearson’s chi-squared or Fisher’s exact test were used for categorical variables. Statistical significance was set at *p* < 0.05.

## Figures and Tables

**Table 1:** Patients profile with ULMS (*n* = 16) *.

Variable	Features	*N* (%)
Menopause	YES	8 (50%)
	NO	6 (37.5%)
Clinical FIGO stage	Tumor restricted to the uterus	8 (50%)
	Pelvic extension	2 (12.5%)
	Extra-pelvic extension	2 (12.5%)
	Visceral metastasis	4 (25%)
Symptoms	Pain	3 (19%)
	Bleeding	6 (37.5%)
	Discharge	0 (0%)
	Pelvic mass	1 (6%)
	No symptoms	2 (12.5%)
Relapse	YES	3 (18.75%)
	NO	6 (37.5%)
	Persistence of disease	3 (18.75%)
Metastasis	YES	4 (25%)
	NO	6 (37.5%)
Death	YES	11 (69%)
	NO	3 (19%)

* Some samples data were ignored.
